# Large overlap in neutrophil transcriptome between lupus and COVID-19 with limited lupus-specific gene expression

**DOI:** 10.1136/lupus-2023-001059

**Published:** 2024-01-31

**Authors:** Rayan Najjar, Noga Rogel, Jose Mario Bello Pineda, Xiaoxing Wang, Megan Tran, Alison Bays, Tomas Mustelin

**Affiliations:** Department of Medicine, University of Washington, Seattle, Washington, USA

**Keywords:** Systemic Lupus Erythematosus, COVID-19, Autoimmune Diseases

## Abstract

**Objectives:**

To illuminate the poorly understood aetiology of SLE by comparing the gene expression profile of SLE neutrophils with that of neutrophils from patients infected by SARS-CoV-2, a disease (COVID-19) with well-defined antigens and a similar type I interferon response.

**Methods:**

RNA sequencing of neutrophils from patients with SLE (n=15) and healthy controls (n=12) was analysed for differential gene expression and modulated pathways. The same analyses were performed on a similar neutrophil dataset from patients with SARS-CoV-2 infection (n=30) and healthy controls (n=8). Next, we carried out comparative analyses to identify common and unique transcriptional changes between the two disease contexts, emphasising genes regulated in opposite directions.

**Results:**

We identified 372 differentially expressed genes in SLE neutrophils compared with healthy donor neutrophils (≥2 fold, p<0.05), 181 of which were concordant with transcriptional changes in SARS-CoV-2-infected individuals compared with their respective healthy controls. In contrast, 118 genes demonstrated statistically significant alterations exclusive to SLE, including 28 genes that were differentially expressed in opposite directions in the two diseases.

**Conclusions:**

The substantial overlap between neutrophil responses in SLE and COVID-19 suggests that the unknown cause of SLE is functionally similar to a viral infection and drives a similar immune activation and type I interferon response. Conversely, the genes regulated in the opposite direction represent responses unique to SLE. These include tyrosylprotein sulfotransferase-1 and nucleic acid deaminases of the APOBEC family, which can catalyse cytosine-to-uridine editing of both RNA and DNA, and other RNA-modifying enzymes.

## Introduction

Neutrophils act in SLE^1^ as sources of immunogenic and proinflammatory nucleic acids (eg, double-stranded DNA) and ribonuclear protein complexes. They also contribute to immune complex-driven inflammation and tissue injury. For example, after skin exposure to ultraviolet light, a trigger of SLE exacerbation, neutrophils migrate to the skin and subsequently to the kidneys, where they initiate tissue damage.^2^

Starting from the observation that SLE is associated with elevated type I interferons similar to viral infections, we compared neutrophils from these two conditions by RNA sequencing, expression profiling and pathway analysis.

## Methods

### Patients with SLE and healthy donors

Adult patients with SLE (n=15) and healthy controls (n=12) were included in our study (table 1). Our procedures for neutrophil isolation, RNA extraction and RNA sequencing were recently published.^3^

**Table 1:** Characteristics of patients with SLE

Characteristic	SLE (n=15) ±SD	HC (n=12) ±SD
Age	42.9±15.2	35.3±15.9
Male/female	2/13	3/9
Laboratory measures		
C3 (g/L)	130.0±24.7	n.d.
C4 (g/L)	27.9±13.8	n.d.
Anti-dsDNA	9 positive/3 negative	n.d.
Disease activity and ACR criteria		
SLEDAI	6.9±6.5 (0–19)	n.d.
SLEDAI ≤4	8	n.d.
SLEDAI ≥6	7	n.d.
ISGs >95th percentile of HC	11	n.d.
ISGs <95th percentile of HC	4	n.d.
Oral ulcers	1	n.d.
Rash	2	n.d.
Alopecia	3	n.d.
Arthritis	5	n.d.
Nephritis	2	n.d.
Pleuritis/pericarditis	1	n.d.
CNS symptoms	2	n.d.
Current treatment		
None		12
Hydroxychloroquine	14	n.d.
Steroid	2	n.d.
Additional DMARD	7	n.d.
Biologic	2	n.d.

ACR, American College of Rheumatology; CNS, central nervous system; DMARD, disease-modifying antirheumatic drug; dsDNA, double-stranded DNA; HC, healthy controls; ISGs, interferon stimulated genes; n.d., not determined; SLEDAI, systemic lupus erythematosus (SLE) Disease Activity Index.

### RNA sequencing analysis

Sequencing reads were mapped to the reference genome, followed by gene quantification and differential analysis for both the SLE and COVID-19 datasets. P values were adjusted for multiple comparisons and statistical significance was set at 0.05. Genes with potential unique expression in SLE were identified by subtracting genes with similar expression in COVID-19 using liberal p value cut-offs (see online supplemental methods).

10.1136/lupus-2023-001059.supp2 Supplementary data

### Gene pathway analysis

Gene Set Enrichment Analysis was performed on ranked lists of all genes using the Hallmark gene sets. Gene Ontology biological process (GO:BP) and molecular function (GO:MF) enrichment analysis used the gprofiler2 package for genes that were differentially expressed (DE) in SLE versus healthy controls, before and after exclusion of genes that were similarly expressed in COVID-19.

### COVID-19 neutrophil datasets

We used neutrophil RNA sequencing (GEO GSE212041)^4^ data from hospitalised COVID-19 patients. We only included samples from the first day of admission from patients with respiratory symptoms and febrile illness who tested positive for COVID-19, did not have severe comorbidities and did not require invasive ventilation. This resulted in 30 COVID-19 patients vs 8 healthy controls.

To reduce errors introduced by differences in methodology, patient sampling or the time course of SARS-CoV-2 infection, we also used data from a different study.^5^ These data consisted of RNA sequencing of neutrophils from patients hospitalised for severe COVID-19. Because this study did not include healthy donors, we restricted our analysis to those patients who eventually were discharged, and used their earliest (acute phase COVID-19; n=4) and last (n=4) samples. We used likelihood ratio test for hypothesis testing using a reduced design formula in DESeq2. We refer to this dataset as ‘acute versus convalescent COVID-19’.

## Results

### Differential gene expression, major pathways and gene expression profiles in SLE neutrophils

Differential gene analysis showed 372 genes with ≥2 fold (346 upregulated, 26 downregulated) in SLE neutrophils compared with healthy donor neutrophils (online supplemental figure 1A).

10.1136/lupus-2023-001059.supp1 Supplementary data

Gene set enrichment analysis in SLE versus healthy control neutrophils showed that nine major pathways were upregulated in SLE with false discovery rate q-values of <0.25 (online supplemental figure 1B,C).

A more detailed functional enrichment analysis of the 346 upregulated genes using Gene Ontology showed enrichment of 473 GO:BP and 56 GO:MF pathways. The top 25 GO:BP pathways by p value are shown in online supplemental figure 1D. The top 25 GO:MF pathways by p value (online supplemental figure 1E) included double-stranded RNA binding and deaminase activity, and other RNA metabolism functions.

All these data are consistent with previous reports^6 7^ and show that neutrophils in patients with SLE are activated in a seemingly broad host defence response reminiscent of antiviral immunity, even though SLE is not generally considered a pathogen-driven disease.

### DE genes and pathways in COVID-19 neutrophils

There were 936 upregulated and 298 downregulated genes at ≥2 fold (p<0.05) in COVID-19 neutrophils compared with controls. Pathway analysis showed seven of the nine pathways upregulated in SLE were also upregulated in COVID-19 (online supplemental figure 2A).

GO:BP pathways were similar to those in SLE neutrophils (online supplemental figure 2D), plus complement and phagocytosis pathways, and fewer virus-related than in SLE. Antigen binding, immunoglobulin receptor binding, lipopolysaccharide binding, caspase and C-C chemokines were represented more than in SLE. These results indicate a broader immune response than in SLE, perhaps related to the more acute inflammation and organ damage in severe COVID-19.

### Direct comparisons between SLE and COVID-19 neutrophils

**Figure 1:**
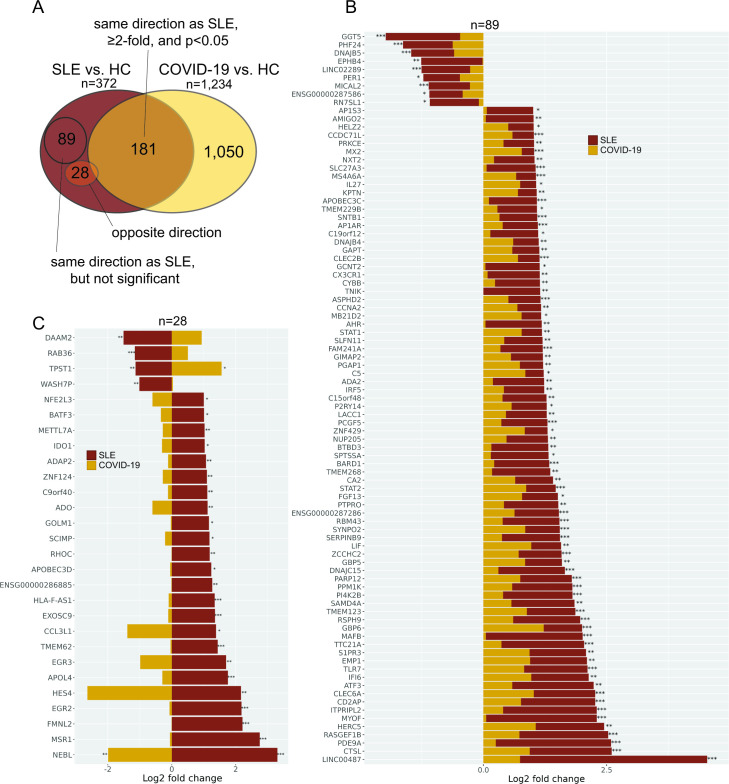
Comparisons of neutrophil gene expression between SLE and COVID-19. (A) Venn diagram of the 372 DE genes in SLE neutrophils and their overlap with COVID-19 genes, as indicated. (B) The 89 genes that are DE in SLE (p<0.05) and expressed in the same direction, but not statistically significant (p≥0.1 or p≥0.2 if fold change >2) in COVID-19. (C) The 28 DE genes in SLE neutrophils (p<0.05) expressed in the opposite direction in COVID-19 neutrophils. Asterisks indicate statistical significance in relation to the relevant healthy controls (HC): *p<0.05, **p<0.01, ***p<0.005. DE, differentially expressed.

To better understand the similarities and differences between SLE and COVID-19 neutrophils, we focused on the DE genes in each condition compared with their respective healthy controls instead of merging the two datasets. This approach avoids batch effects that often exist between datasets from different laboratories, as well as confounding effects on individual genes by batch effect correction tools. Of the 372 genes significantly DE in SLE, 181 were also ≥2 fold DE (p<0.05) in the same direction in SARS-CoV-2-infected individuals compared with their respective healthy controls (figure 1A). Another 89 genes were statistically significantly altered in SLE, but not in COVID-19 (figure 1), although they showed change in the same direction. 28 genes were DE in opposite directions in the two diseases (figure 1), but only two of them were statistically significant also in COVID-19 (*TPST1* and *NEBL*).

**Figure 2:**
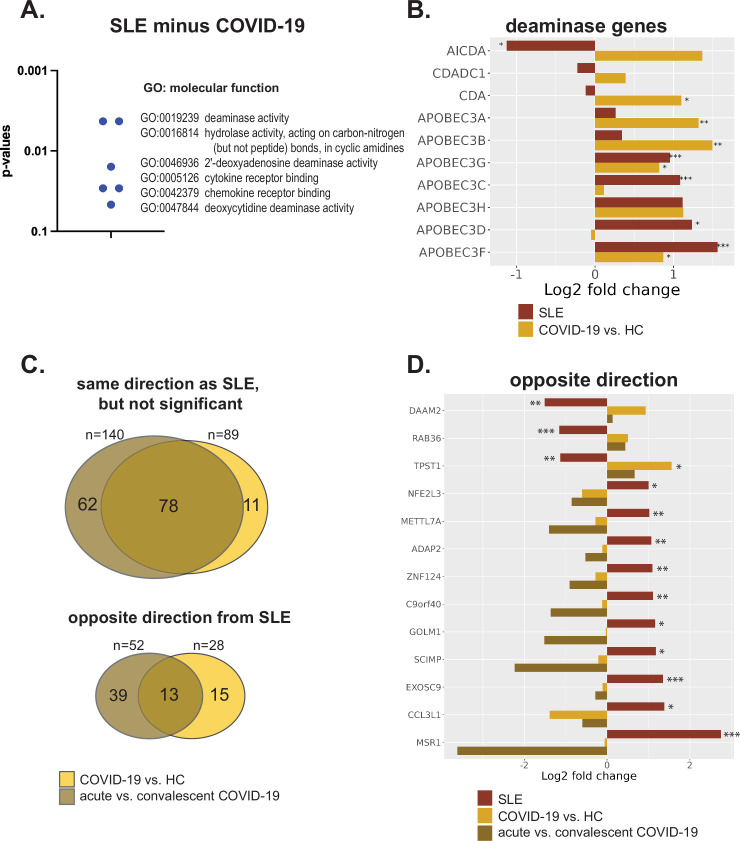
Comparisons of neutrophil gene expression between SLE and COVID-19. (A) The six statistically significant GO molecular function enriched gene sets in SLE data subtracted with the COVID-19 data. (B) Expression of the 10 genes of the deaminase pathways in SLE and COVID-19. (C) Venn diagram (upper) depicting the DE genes in SLE neutrophils expressed in the same direction, but far from being statistically significant (p≥0.1 or p≥0.2 if fold change >2) in one or both of the COVID-19 data, as indicated. Venn diagram (lower) depicting the DE genes in SLE neutrophils expressed in the opposite direction in one or both of the COVID-19 data, as indicated. (D) The 13 DE genes in SLE neutrophils (p<0.05) expressed in the opposite direction in both the first COVID-19 and the convalescent COVID-19 data. Asterisks indicate statistical significance in relation to the relevant healthy controls (HC): *p<0.05, **p<0.01, ***p<0.005. DE, differentially expressed; GO, Gene Ontology.

To identify pathways unique to SLE, we performed Gene Ontology analysis of the genes that differed between SLE and COVID-19 (ie, the 89 and 28 genes in figure 1). This yielded 62 statistically significantly enriched GO:BP gene sets (similar to those in SLE; not shown) and 6 statistically significant GO:MF sets (figure 2A), including 3 with nucleic acid deaminase activity unique to SLE. Genes in this pathway, *AICDA, CDADC1, CDA* and *APOBEC3D,* were regulated in opposite directions (only *AICDA* was statistically significantly downregulated in SLE and *CDA* up in COVID-19), while *APOBEC3A* and *APOBEC3B* were significantly upregulated in COVID-19, but not SLE. *APOBEC3C* and *APOBEC3G* were statistically significantly upregulated only in SLE (figure 2B).

### Validation in a second COVID-19 cohort

Of the 372 DE genes in SLE, 110 genes had same-direction DE in this second COVID-19 cohort (≥2 fold, p<0.05). Of the 89 genes only DE in SLE, but not the first COVID-19 cohort, 78 genes were shared with acute versus convalescent COVID-19 (figure 2C). Lastly, of the 28 genes modulated in opposite directions in SLE and the first COVID-19 cohort, 13 genes had the same pattern in the acute versus convalescent COVID-19 neutrophil comparison (figure 2C,D). We consider these 13 to be the most likely to hold keys to the uniqueness of SLE disease.

## Discussion

The similarity in neutrophil gene expression between COVID-19, caused by a virus, and SLE with an unknown aetiology suggests that the nature of this unknown aetiology must share some features with a viral infection. Gene sets involved in antiviral defence ranked even higher in SLE than in COVID-19. This would be compatible with a role for nucleic acid species that trigger RNA and/or DNA sensors in SLE. Such nucleic acids could arise by covalent modifications like cytosine deamination or adenine methylation. In addition, by contrasting gene expression to a viral condition, we found gene expression changes in SLE beyond the interferon signature; these are more likely to be related to unique SLE biology.

Most type I interferon-inducible genes were elevated in similar patterns in both SLE and COVID-19, with the exception of *TLR7*, which was DE only in SLE. Other exceptions include members of the apolipoprotein B mRNA editing enzyme catalytic subunit 3 (APOBEC3) of cytidine deaminases. SLE neutrophils expressed *APOBEC3C*, *APOBEC3F* and *APOBEC3G*, at elevated levels compared with controls as previously reported.^8^ In contrast, *APOBEC3A* and *APOBEC3B* were higher in COVID-19. It has been suggested that elevated APOBEC3 in SLE may generate point mutations in mRNA that are subsequently translated into neoantigens.^9^ The primary physiological role of most members of the APOBEC3 family is in the defence against retroviruses like HIV and endogenous retroviruses,^10 11^ as well as retrotransposons.^12^ The latter two categories of transposable elements are more active in SLE neutrophils than in healthy neutrophils.^3 13 14^

We have varying levels of confidence in the uniqueness of the identified genes to SLE. Genes with significant opposite-direction expression in SLE versus COVID-19 have the most evidence. Only two genes (*TPST1* and *NEBL2*) fulfilled this criterion, and only one (*TPST1*) considering both COVID-19 datasets. *TPST1* encodes tyrosylprotein sulfotransferase-1, an enzyme located in the Golgi adding O-sulfonyl groups to tyrosine residues in transmembrane and extracellular proteins. Still, other genes that were altered in SLE and non-significant in COVID-19 can potentially be meaningful and unique in lupus biology, especially that we used liberal p value cut-offs in COVID-19 to exclude more genes from the SLE-specific list, prioritising specificity, and particularly when similar patterns of expression were also seen in the validation of the COVID-19 dataset.

In summary, we report that SLE neutrophils express genes that largely overlap with genes induced in neutrophils during SARS-CoV-2 infection. Additionally, we identified unique gene expression in SLE, including genes involved in nucleic acid modifications and turnover, that will require further investigation. Atypical endogenous nucleic acid species could trigger DNA or RNA sensor activation leading to type I interferon production and lupus pathogenesis.
