# Supplementary material for: Large overlap in neutrophil transcriptome between lupus and COVID-19 with limited lupus-specific gene expression
Source: Lupus Sci Med. 2024 Jan 31;11(1):e001059. doi: 10.1136/lupus-2023-001059 (PMC10831459; doi:10.1136/lupus-2023-001059)
Supplement: Supplementary data [file lupus-2023-001059supp002.pdf]

# Supplemental Methods

## RNA-Seq analysis

After QC, reads from FASTQ files were mapped to the Genome Reference Consortium Human Build 38 (GRCh38) using STAR aligner (2.7.9a)[1] to generate BAM files. Uniquely mapped reads were assigned to genomic features (e.g. genes, RNA genes) using featureCounts from the Rsubread R package (2.10.5)[2]. We used DESeq2 (1.36.0)[3] to conduct differential gene expression analysis for both the SLE and COVID-19 datasets. Genomic features with low counts (<20) across samples were removed. These genes with very low expression have zero to little chance to contribute to differential expression and removing them improves statistical power. DESeq2 output includes gene-level log2 fold change in expression between study groups and p values adjusted for multiple comparisons. Statistical significance was set at a cutoff of alpha=0.05.

## Comparison between SLE and COVID-19

Instead of combining data from the SLE and COVID-19 diseases, we chose to analyze them separately then compare the results based on log2fc and adjusted p values. This is because existing batch-effect adjustment methodologies will modify the data and carry a risk of altering the biological effects in unexpected ways[6]. In contrast, analyzing the data separately preserves the internal validity of the comparisons between each disease state and its own healthy control samples.

After detection of genes with ≥2 fold statistically significant differentially expressed genes in SLE vs healthy neutrophils, we subset this list further by removing genes with lower gene expression (median normalized gene expression in SLE <20) and keeping genes with 25$^{th}$ quantile in SLE

<minimum or 75th quantile in SLE > maximum of normalized counts in healthy neutrophils to only identify genes with robust relative differences that are less likely to be influenced by outliers. We considered genes in the COVID-19 data to have similar expression to SLE if they had ≥2 fold same-direction change and adjusted p value <0.2, or <2 fold same-direction change and adjusted p value <0.1. We used these liberal p values cutoffs to allow exclusion of more genes from the SLE list, resulting in more confidence in detection of genes with unique differential expression in SLE (i.e. we prioritized specificity). These genes with similar expression in SLE and COVID-19 were excluded from the list of statistically significant differentially expressed genes in SLE vs healthy neutrophils, to arrive at a final list of genes that are more likely to be uniquely expressed in SLE. Additionally, we also identified a subset of genes with high likelihood of having the same type of expression in SLE and COVID-19 (two fold same-direction change and p value <0.05 in both datasets).

1 Dobin, A. *et al.* STAR: ultrafast universal RNA-seq aligner. *Bioinformatics* **29**, 15-21, doi:10.1093/bioinformatics/bts635 (2013).
2 Liao, Y., Smyth, G. K. & Shi, W. The R package Rsubread is easier, faster, cheaper and better for alignment and quantification of RNA sequencing reads. *Nucleic Acids Res* **47**, e47, doi:10.1093/nar/gkz114 (2019).
3 Love, M. I., Huber, W. & Anders, S. Moderated estimation of fold change and dispersion for RNA-seq data with DESeq2. *Genome Biol* **15**, 550, doi:10.1186/s13059-014-0550-8 (2014).
4 Subramanian, A. *et al.* Gene set enrichment analysis: a knowledge-based approach for interpreting genome-wide expression profiles. *Proc Natl Acad Sci U S A* **102**, 15545-15550, doi:10.1073/pnas.0506580102 (2005).
5 Kolberg, L., Raudvere, U., Kuzmin, I., Vilo, J. & Peterson, H. gprofiler2 -- an R package for gene list functional enrichment analysis and namespace conversion toolset g:Profiler. *F1000Res* **9**, doi:10.12688/f1000research.24956.2 (2020).
6 Zhang, Y., Parmigiani, G. & Johnson, W. E. ComBat-seq: batch effect adjustment for RNA-seq count data. *NAR Genom Bioinform* **2**, lqaa078, doi:10.1093/nargab/lqaa078 (2020).
