# Supplementary material for: Large overlap in neutrophil transcriptome between lupus and COVID-19 with limited lupus-specific gene expression
Source: Lupus Sci Med. 2024 Jan 31;11(1):e001059. doi: 10.1136/lupus-2023-001059 (PMC10831459; doi:10.1136/lupus-2023-001059)
Supplement: Supplementary data [file lupus-2023-001059supp001.pdf]

Supplemental Fig. S1

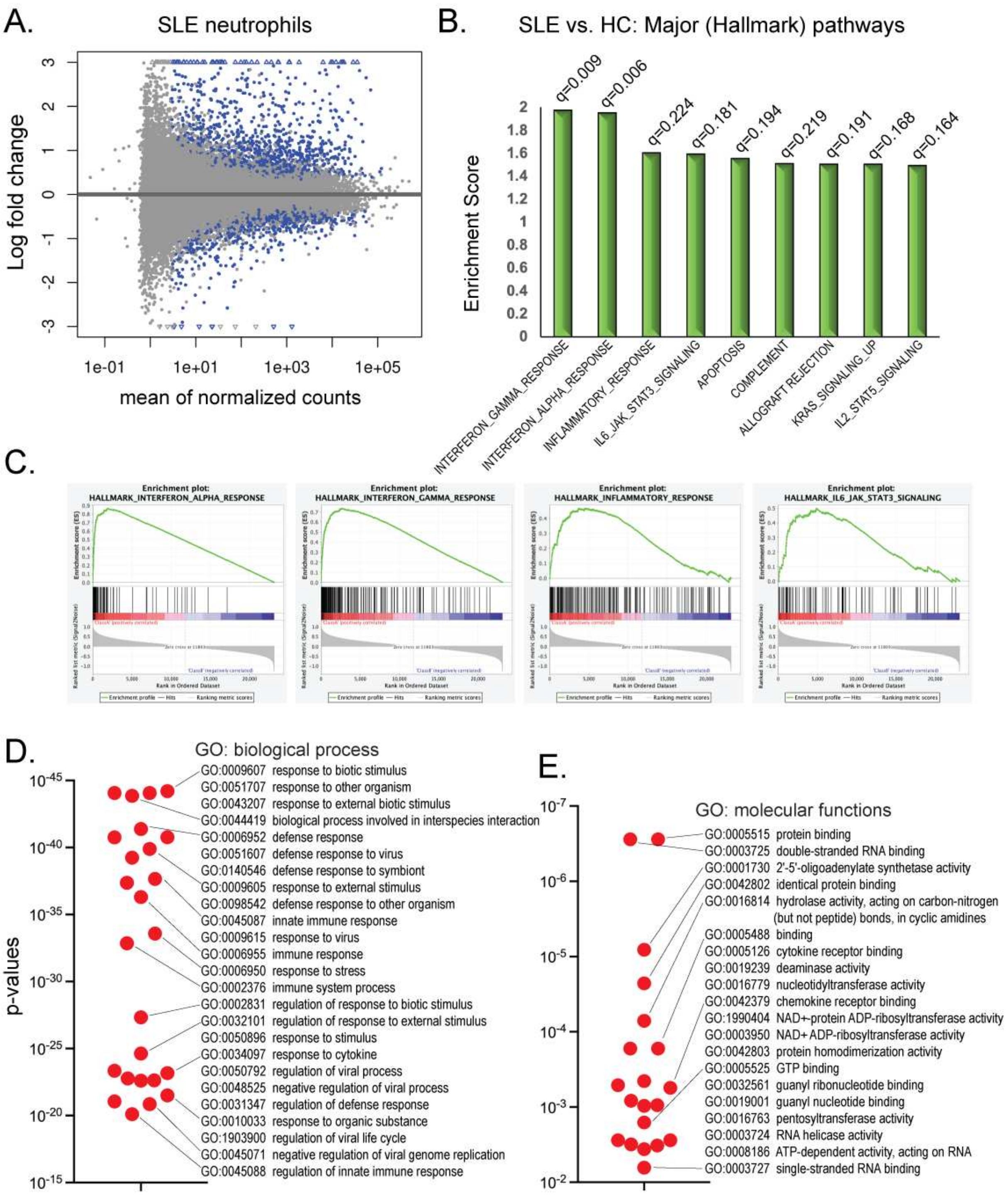

Supplemental Fig. S1 | DE genes in SLE neutrophils compared to healthy controls. A., Volcano plot of DE genes in SLE neutrophils compared to HC neutrophils. Blue dots represent statistically significant genes, blue open triangles values that are higher than log2fc =3, blue open upside-down triangles are less than log2fc =-3. **B.,** Major (Hallmark) pathways statistically significantly upregulated in SLE neutrophils compared to HC neutrophils with the FDR q-value for each. **C.,** Enrichment plots of the 4 top pathways. **D.,** Top 25 (by p-value) Gene Ontology (GO) biological process (GO:BP) enriched gene sets in SLE neutrophils compared to HC neutrophils. **E.,** Top 25 (by p-value) GO molecular function (GO:MF) enriched gene sets in SLE neutrophils compared to HC neutrophils.

Supplemental Fig. S2

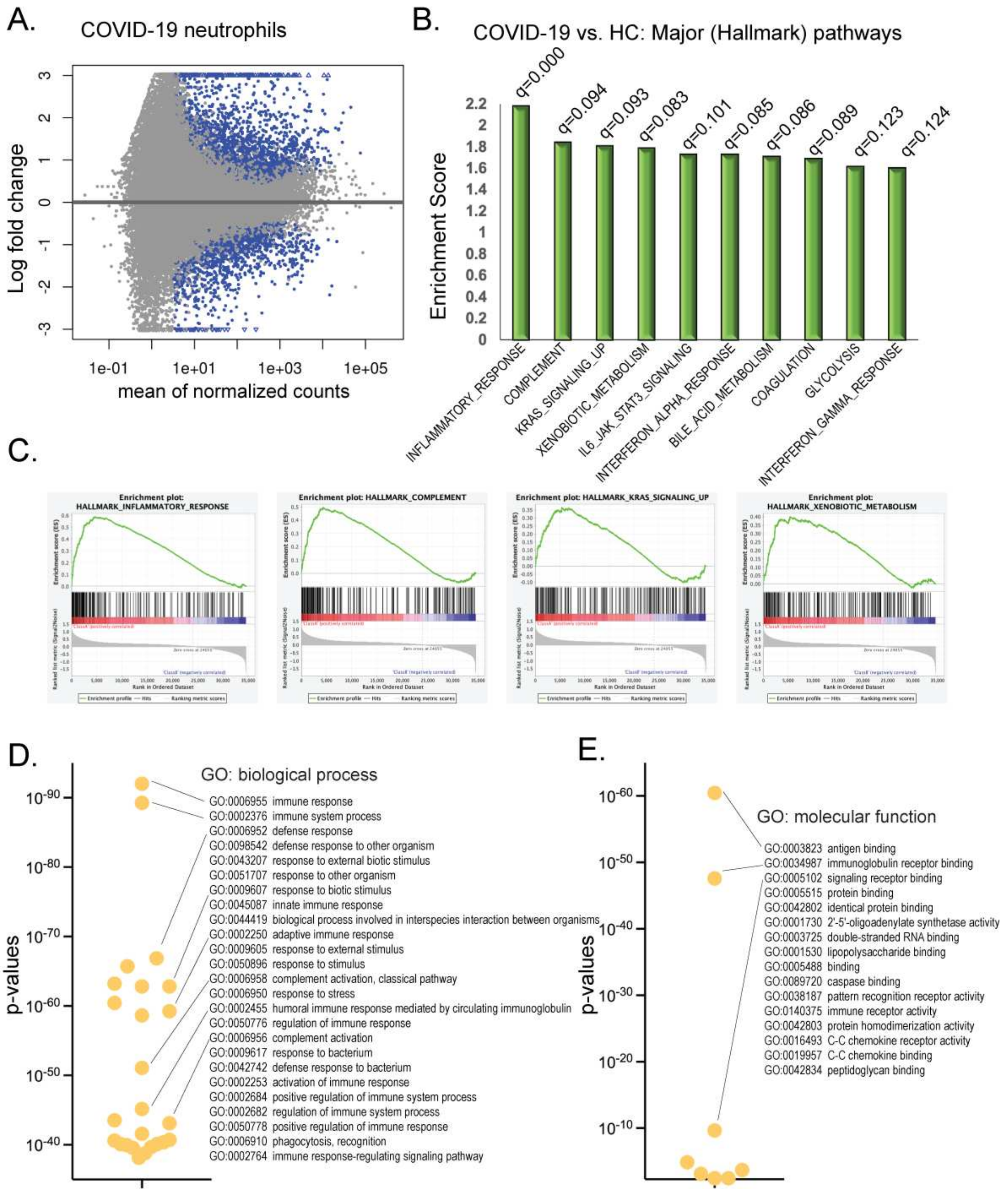

Supplemental Fig. S2 | DE genes in COVID-19 neutrophils compared to their healthy controls. A., Volcano plot of DE genes in COVID-19 neutrophils compared to HC neutrophils and their overlap. Blue dots represent statistically significant genes, blue open triangles values represent log2fc >3, blue open upside-down triangles are log2fc <-3. **B.,** Major (Hallmark) pathways statistically significantly upregulated in COVID-19 neutrophils compared to HC neutrophils with the FDR q-value for each. **C.,** Enrichment plots of the 4 top pathways. **D.,** Top 25 (by p-value) Gene Ontology (GO) biological process (GO:BP) enriched gene sets in COVID-19 neutrophils compared to HC neutrophils. **E.,** The 8 statistically significant GO molecular function (GO:MF) enriched gene sets in COVID-19 neutrophils compared to HC neutrophils.
